# Environmental Tobacco Smoke Exposure during Pregnancy and Child Neurodevelopment

**DOI:** 10.3390/ijerph14070796

**Published:** 2017-07-17

**Authors:** Kinga Polanska, Anna Krol, Dorota Merecz-Kot, Danuta Ligocka, Karolina Mikolajewska, Fiorino Mirabella, Flavia Chiarotti, Gemma Calamandrei, Wojciech Hanke

**Affiliations:** 1 Department of Environmental Epidemiology, Nofer Institute of Occupational Medicine, 91-348 Lodz, Poland; anna.krol@imp.lodz.pl (A.K.); wojciech.hanke@imp.lodz.pl (W.H.); 2 Department of Health and Work Psychology, Nofer Institute of Occupational Medicine, 91-348 Lodz, Poland; merecz@imp.lodz.pl; 3 Bureau of Quality Assurance, Nofer Institute of Occupational Medicine, 91-348 Lodz, Poland; danuta.ligocka@imp.lodz.pl; 4 Department of Biological and Environmental Monitoring, Nofer Institute of Occupational Medicine, 91-348 Lodz, Poland; karolina.mikolajewska@imp.lodz.pl; 5 Center for Behavioral Sciences and Mental Health, National Institute of Health, I-00161 Rome, Italy; fiorino.mirabella@iss.it (F.M.); flavia.chiarotti@iss.it (F.C.); gemma.calamandrei@iss.it (G.C.)

**Keywords:** environmental tobacco smoke, passive smoking, pregnancy, prenatal period, child neurodevelopment, cognitive, language and motor functions

## Abstract

The developing fetus is especially vulnerable to environmental toxicants, including tobacco constituents. The aim of this study was to assess the impact of environmental tobacco smoke (ETS) exposure during pregnancy on child neurodevelopment within the first two years of life. The study population consisted of 461 non-smoking pregnant women (saliva cotinine level <10 ng/mL). Maternal passive smoking was assessed based on the cotinine level in saliva analyzed by the use of high-performance liquid chromatography coupled with tandem mass spectrometry (HPLC-ESI + MS/MS) and by questionnaire data. The cotinine cut-off value for passive smoking was established at 1.5 ng/mL (sensitivity 63%, specificity 71%). Psychomotor development was assessed in children at the age of one- and two-years using the Bayley Scales of Infant and Toddler Development. Approximately 30% of the women were exposed to ETS during pregnancy. The multivariate linear regression model indicated that ETS exposure in the 1st and the 2nd trimesters of pregnancy were associated with decreasing child language functions at the age of one (β = −3.0, *p* = 0.03, and β = −4.1, *p* = 0.008, respectively), and two years (β = −3.8, *p* = 0.05, and β = −6.3, *p* = 0.005, respectively). A negative association was found for cotinine level ≥1.5 ng/mL in the 2nd trimester of pregnancy and child cognition at the age of 2 (β = −4.6, *p* = 0.05), as well as cotinine levels ≥1.5 ng/mL in all trimesters of pregnancy and child motor abilities at two years of age (β = −3.9, *p* = 0.06, β = −5.3, *p* = 0.02, and β = −4.2, *p* = 0.05, for the 1st, the 2nd, and the 3rd trimester of pregnancy, respectively; for the 1st trimester the effect was of borderline statistical significance). This study confirmed that ETS exposure during pregnancy can have a negative impact on child psychomotor development within the first two years of life and underscore the importance of public health interventions aiming at reducing this exposure.

## 1. Introduction

While the impact of prenatal exposure to tobacco, due to active maternal smoking, on child neurodevelopment is well established, the association between maternal environmental tobacco smoke (ETS) exposure during pregnancy and child development has been studied less frequently [1]. This issue is of great importance, as a large proportion of women are exposed to passive smoking. 

During prenatal development the nervous system may be more susceptible to the negative effects of environmental toxicants, including tobacco constituents [2]. Changes in the brain can be associated with neurodevelopmental outcomes, such as reduced psychomotor functions and increased risk of behavioral problems. The possible mechanism involves fetal hypoxia as a result of reduced utero-placental blood flow and reduced oxygen carrying capacity of blood by the increased carboxyhemoglobin levels [2,3,4,5]. In addition, influence on neurotransmitter function by targeting nicotine acetylcholine receptors [2,6], upregulation of nicotinic cholinergic receptor binding sites [2,7], as well as modification of monoaminergic synaptic function [8,9,10] need to be mentioned. Finally, tobacco smoke contains many other toxic chemicals, including cadmium and lead, which can affect the developing nervous system.

The negative impact of ETS exposure during pregnancy on child neurodevelopment including the risk of developmental delay has been reported in a few studies [11,12,13]. In addition, some studies suggest that maternal passive smoking in pregnancy may impact child behavioural development, particularly aggressive and externalizing behaviours [14,15,16,17]. On the other hand, Lee and co-workers have indicated no impact of prenatal ETS exposure on the psychomotor developmental index score [2], and Roza and co-workers [18] have pointed out that the statistical association between parental smoking and behavioural problems was strongly confounded by parental characteristics, mainly socio-economic status and psychopathology. 

In Poland, based on the Global Adult Tobacco Survey that was conducted between the years 2009 and 2010 about 30% of the non-smoking women were exposed to tobacco smoke at home [19]. Our previous assessments that covered 1771 women from the Polish Mother and Child Cohort (REPRO_PL) have indicated that about 15% of them can be classified as active, and about 35% as passive smokers according to their cotinine level in saliva [20]. We have also noted adverse effects of maternal active smoking during pregnancy on child motor development [21,22].

In view of the above, we hypothesized that environmental tobacco smoke (ETS) exposure during pregnancy negatively affects child cognitive, language and motor abilities at the age of 1 and 2 years, and we aimed at verifying this hypothesis using the data from the Polish Mother and Child Cohort (REPRO_PL). To test the hypothesis the exposure (ETS), outcome (child neurodevelopment at age one and two years), and confounding variables were defined and appropriate data were collected. 

## 2. Material and Methods

### 2.1. Study Design and Population

The detailed description of the REPRO_PL cohort and its methodology has been published previously [23,24]. Briefly, this multicentre prospective study was established in 2007, covering healthy women (according to the criteria specified in the study protocol) in single, healthy pregnancy of up to 12 weeks of gestation (with no assisted conception and no pregnancy complications). 

The women were interviewed by a trained midwife once in each trimester of pregnancy in order to obtain socio-demographic characteristics, medical and reproductive history, as well as information on the environmental/lifestyle and occupational exposure. 

During each visit and after delivery, biological samples (including saliva, urine, blood, hair, and cord blood) were collected [23].

When the child achieved one year of age his/her exposure, health status, and neurodevelopment was assessed by a paediatrician/allergist and a developmental psychologist [24]. A similar procedure was repeated two years after birth.

In total, 539 children were examined at least once within two years after their birth (303 children had both examinations at one and two years of age, 198 were examined only at around the 12th month of age, and 38 only at the 24th month of age) [25]. Taking into account the fact that the current study is aimed at the assessment of the impact of maternal ETS exposure during pregnancy on child neurodevelopment, the smokers were excluded from the analysis and the study sample consisted of 461 (85.5% of the study population) mother-child pairs. 

The study was approved by the Ethical Committee of the Nofer Institute of Occupational Medicine (NIOM), Lodz, Poland (Nos. 7/2007 and 3/2008) and written consent was obtained from all of the study subjects. 

### 2.2. Exposure Variables

Based on existing knowledge and our previous estimates there is no gold standard for assessment of ETS exposure [20]. Thus, in our study, prenatal ETS was measured by assessing mother’s cotinine levels in saliva during pregnancy and, additionally, by collecting (at the same time as the sample collection) the questionnaire information about their husband/partner smoking and smoking allowance at home.

At enrolment (the 1st trimester of pregnancy) and each follow-up visit (the 2nd and 3rd trimesters of pregnancy) the women provided saliva samples into a salivette with citric acid (Sarstedt, Nümbrecht, Germany). A detailed description of the sample collection and analysis has been published elsewhere [20,26]. Briefly, the cotinine concentration in saliva was analysed at NIOM using high-performance liquid chromatography coupled with tandem mass spectrometry/positive electrospray ionisation (HPLC-ESI + MS/MS) and an isotope dilution method (method accredited by the Polish Centre of Accreditation: Certificate AB 215), with a limit of detection (LOD) of 0.12 ng/mL and a limit of quantification (LOQ) of 0.4 ng/mL [20]. Based on the previous assessments, the cut-off points of 10 ng/mL (sensitivity 96%, specificity 95%) for active smoking and 1.5 ng/mL (sensitivity 63%, specificity 71%) for passive smoking were selected [20]. As it was mentioned above, smokers (the women with cotinine level ≥10 ng/mL) were excluded from the analysis. The independent variable was considered as dichotomous (not exposed to ETS: cotinine <1.5 ng/mL vs. exposed to ETS: cotinine ≥1.5 ng/mL). 

At the time of saliva collection the women filled in questionnaires that covered two separate questions about ETS exposure: (1) “Does your husband/partner smoke cigarettes?” and (2) “Is smoking allowed at your home?” (with the possible answers: yes; no). 

The final model evaluates the impact of child prenatal exposure to ETS based on three variables (cotinine level, husband/partner smoking, and smoking allowance at home) on child psychomotor development.

### 2.3. Outcome Variables

The Bayley Scales of Infant and Toddler Development (Bayley 3rd edition) was used to assess children’s neurodevelopment at one and two years of age. Details regarding child psychomotor assessment have been published elsewhere [21,22,24]. In the analysis child cognitive, language, and motor functions were evaluated. 

### 2.4. Counfounding Variables

The following potential confounders were evaluated for the inclusion as covariates in the regression models: maternal age (at child birth) and education (below high school; high school; university degree), marital status (married; unmarried), socio-economic status (SES: low; medium; high), child sex, major pregnancy complications which appeared after inclusion into the study, type of delivery (vaginal; caesarean), gestational age and birth outcomes, breastfeeding (yes/no and duration of breastfeeding), number of siblings, day care attendance, alcohol consumption during pregnancy, and child ETS exposure within the first two years of life. 

SES of the family was measured based on the following question: “What is the financial status of your family?” Women who declared that they have sufficient money for current expenses and that it is possible for them to put a substantial sum aside were allocated into the high income category. Those who indicated sufficient money for current expenses, with possibility to put aside some money were allocated into the medium category, and those who declared insufficient money for current expenses into the low income category.

Child ETS exposure after birth was evaluated based on the maternal declaration about her smoking status within one and two years after delivery (yes; no). Additional analysis was performed with ETS exposure based on cotinine levels in urine collected from the children at one and two years of age (taking into account the difficulties in urine collection at young age the urine samples were not available for all children). The cotinine level was assessed using the same method as described above (with LOD of 0.05 ng/mL, and LOQ of 0.8 ng/mL). Details regarding covariates assessment have been described elsewhere [27]. 

All the confounders with *p* < 0.10 were included in the final models.

### 2.5. Statistical Analysis 

As it was already specified, our hypothesis was that ETS exposure negatively affected children’s psychomotor development in the first two years of life. If the null hypothesis of no relationship between exposure and outcome of interest is rejected, then it can be concluded that such a relationship exists (alternative hypothesis) with a small Type I error probability. As the first step of analysis, frequencies for qualitative variables and means ± SD for quantitative variables were calculated. Then we used the Spearman correlation coefficient to examine the correlation pattern among variables (either exposure variables or psychomotor developmental scores) and we selected the potential confounding variables (covariates) to be included in the subsequent regression analyses (based on criteria described in Section 2.4). Successively, we tested our hypothesis by performing multivariate linear regression, with cognitive, language, and motor developmental scores at one and at two years of age as dependent variables in separate analyses, and ETS exposure based on the cotinine level in the mother’s saliva during different periods of pregnancy as independent variables. The following covariates were included in the initial models: examiner, mother’s age and education, child sex, and SES. By additional multivariate regression analyses we simultaneously verified the effect of ETS exposure measured by the cotinine level in the mother’s saliva during different periods of pregnancy (as above) along with husband smoking status and smoking allowance at home during pregnancy, and child ETS exposure after birth based on maternal smoking status. Finally, two additional analyses were performed and presented in Supplementary Materials: (1) with ETS exposure in the first or second year of life based on cotinine level in child urine (as potential confounder), and (2) regression analysis stratified by SES. 

The variance inflation factor (VIF) was computed for any variable in each model to verify the presence of multicollinearity among explanatory variables; in the case of VIF values higher than 4 the corresponding independent variables would be excluded from the model. 

Regression coefficients are reported together with their level of statistical significance (*p*-value). SPSS (Statistical Package for Social Science, McGraw-Hill Inc., New York, NY, USA) software was used for the statistical analyses.

## 3. Results

### 3.1. Maternal and Child Characteristics

Maternal and child characteristics are summarized in Supplementary Materials (Table S1). A high percentage of the women were married (78%), had a university degree (68%), and represented medium or high SES (90%). 

The mean composite scores for cognitive, language and motor development were on an average or a high average level (Table S2). A positive correlation was observed for each subscale of the Bayley test results for one year assessment (*p* < 0.01) and between cognitive and language, as well as motor and language functions (*p* < 0.01) for two-year assessment (Table 1). The correlation was weaker for comparisons performed between one and two years of age.

### 3.2. Characteristics of Pre- and Postnatal ETS Exposure

Characteristics of the exposure variables are presented in Supplementary Materials (Table S2). Less than 7% of the values for the cotinine level in maternal saliva were below the LOQ. More than 30% of the women could be classified as exposed to ETS based on their cotinine level in saliva collected in each trimester of pregnancy. The same percentage of the women indicated that their husbands/partners smoked cigarettes when they were pregnant (30% in the 1st trimester). This proportion slightly decreased in the 3rd trimester of pregnancy (27%). Even fewer women reported that smoking was allowed at their home (21% in the 1st and 17% in the 3rd trimester of pregnancy).

The cotinine levels in each trimester of pregnancy were highly correlated; *p* < 0.01 (Table 2). Significant, but not strong, correlations were observed between the cotinine levels and husband/partner smoking status for the 1st and 2nd trimester of pregnancy (*p* < 0.05). Correlations between cotinine levels and smoking allowance at home were 0.2 for all trimesters of pregnancy (*p* < 0.01). High correlations were identified between observations concerning husband/partner smoking status as well as smoking allowance at home in the different trimesters of the women’s pregnancy (r > 0.8 and r > 0.7 respectively; *p* < 0.01). 

About 10% of the mothers indicated tobacco smoking within one year after their child birth. More than 30% of the children were exposed to ETS because of household members smoking status. Similar percentages were observed for the period between one and two years of the children’s age (for maternal smoking within one and two years after delivery: correlation coefficient r > 0.6; for household smoking status within one and two years after their child birth: r > 0.7; *p* < 0.01 (Table 2).

### 3.3. Association between ETS Exposure during Pregnancy Based on Cotinine Level in Maternal Saliva and Child Neurodevelopment

The multivariate linear regression model indicated a statistically significant negative association between ETS exposure in the 1st and 2nd trimesters of pregnancy (cotinine level ≥1.5 ng/mL) and child language development at the age of one and two years (*p* ≤ 0.03) (Table 3). In addition, passive smoking in the 2nd trimester of pregnancy was associated with reduced cognitive development among two-year-old children (*p* = 0.01). Prenatal ETS exposure had also a negative impact on child motor abilities in the assessments performed at the age of two years (*p* ≤ 0.02). 

### 3.4. Association between ETS Exposure during Pregnancy Based on Cotinine Level in Maternal Saliva, Husband/Partner Smoking Status, and Smoking Allowance at Home and Child Neurodevelopment

The final model included the same confounding variables mentioned above, with the addition of child ETS exposure after birth (based on maternal smoking status), and all three indicators of prenatal ETS exposure (dichotomized: cotinine level, husband smoking and smoking allowance at home) (Table 4). This analysis confirmed all the associations observed in previous regression analyses. ETS exposure in the 1st and the 2nd trimesters of pregnancy was associated with reduced child language functions at the age of one (β = −3.0, *p* = 0.03 and β = −4.1, *p* = 0.008, respectively) and two years (β = −3.8, *p* = 0.05 and β = −6.3, *p* = 0.005, respectively). A negative association was found for the cotinine level ≥1.5 ng/mL in the 2nd trimester of pregnancy and child cognition at the age of two years (β = −4.6, *p* = 0.05), as well as cotinine levels ≥1.5 ng/mL in all trimesters of pregnancy and child motor abilities at two years of age (β = −3.9, *p* = 0.06, β = −5.3, *p* = 0.02, and β = −4.2, *p* = 0.05, for the 1st, the 2nd, and the 3rd trimester of pregnancy, respectively; for the 1st trimester the effect was of borderline statistical significance). In addition, smoking husband/partner when the women was pregnant had a significant negative impact on child cognitive development at the age of one year (β = −3.3, *p* = 0.02; β = −3.5, *p* = 0.05; β = −3.7; *p* = 0.02 for the 1st, the 2nd and the 3rd trimester, respectively).

Additional regression analysis was performed on a subset of children, using cotinine in their urine as potential confounders in the model (Table S3). Results of this analysis are in substantial accordance with those presented in Table 4, thus supporting the reliability of the questionnaire data in measuring the postnatal exposure to smoking.

### 3.5. Association between ETS Exposure during Pregnancy Based on the Cotinine Level in Maternal Saliva and Child Neurodevelopment—Analysis Stratified by Maternal SES

The analysis stratified by maternal SES (low/medium and high) is presented in Supplementary materials (Tables S4 and S5). Associations between exposure and child neurodevelopment were roughly comparable in the two strata, except for the language domain, where high SES exerts a protective role on the development likely overshadowing the effect of other risk factors. However, significant effects of exposure were observed only in the low/medium SES group, likely due to the larger sample size.

## 4. Discussion

The present study showed that 30% of the non-smoking women were exposed to ETS during the pregnancy period. Children of the mothers who were passive smokers while pregnant had decreased neurodevelopmental abilities compared with children of the non-exposed mothers. This association was observed for the whole spectrum of child neurodevelopment, including cognitive, language, and motor abilities.

In the studies assessing ETS exposure on early development of children, a reliable assessment of passive smoking and psychomotor abilities is the most challenging issue. In the current study we used three indicators of ETS exposure, i.e., cotinine levels in saliva, husband/partner smoking status, and allowance of smoking at home. It seems that the cotinine level can give a better estimate of exposure than the questionnaire data [28,29,30,31]. ETS assessment based on a husband/partner’s smoking status and exposure at home or in public places might lead to a misclassification, especially during the pregnancy period. The existing studies in this field have selected a variety of indicators of ETS exposure including: the number of cigarettes smoked daily in the presence of a mother over the period of pregnancy [13], at least one person that smoked in a woman’s environment for at least 30 minutes [14], paternal smoking outdoors and indoors [18], ever smelt tobacco smoke at home, in the workplace and/or outdoors [2], or the cotinine level in umbilical cord blood [12]. Based on our previous assessment (on the REPRO_PL cohort) nearly 30% of the non-smokers who indicated that smoking was not allowed at their home could be classified as exposed to passive smoking based on the cut-off value [20]. The differences in the methods selected for exposure assessment can lead to differences between the studies with respect to the percentages of women exposed to ETS during pregnancy and the strength of the association between the exposure of interest and child neurodevelopment. The proportion of the women exposed to ETS in our study is in agreement with the data from a representative survey conducted in Poland at a similar time point (30% of the non-smoking women exposed to ETS at home). A similar percentage (37%) of women exposed to ETS has been reported by Liu et al. [14]. A slightly lower percentage of newborns exposed to ETS in the prenatal period based on the cord blood cotinine has been observed in the Taiwan Birth Panel Study (25%) [12]. In the study by Roza et al. among never smokers, paternal smoking has been indicated by 32% of the study women and of them those who gave up smoking before pregnancy constituted 44% [18]. A considerably higher percentage of passive smokers in the period of pregnancy has been observed in Korea (64%) [2].

Several reviews suggest associations between maternal smoking during pregnancy (including active and passive smoking) and reduced child psychomotor abilities, as well as behavioural problems. They also underline the importance of controlling for a variety of confounding factors [3,32,33,34,35,36]. In the study conducted in South Korea (Seoul, Cheonan, and Ulsan) and in Poland (Krakow) child psychomotor development has been assessed using the Bayley Scales of Infant Development (BSID-II)—the earliest version of the test, which was used in the current study [2,13]. Similarly to our assessments, they have found a negative impact of prenatal ETS exposure on the Child Mental Development Index score (as a combination of early cognitive and language development) [37]. On the contrary, Lee and co-workers have not observed any impact of the exposure of interest on the child Psychomotor Developmental Index score [2]. 

A Taiwanese cohort study has reported a negative association between cotinine levels and two-year-old children’s neurodevelopment assessed by the developmental quotients of the whole test and cognitive, language, fine motor, and social subtests of the Comprehensive Developmental Inventory for Infants and Toddlers [12]. Those authors have concluded that CYP1A1 Ile462Val and GSTT1 metabolic genes can modify the effect of cord blood cotinine on early child neurodevelopment, especially in terms of language and fine motor development. Rauh and co-workers have concluded that prenatal exposure to ETS has a negative impact on two-year-olds’ cognitive development, an effect that is exacerbated under conditions of material hardship [11]. Differences in those studies’ findings can be related to the differences in exposure and outcome assessments (different sensitivities of the selected measures), child age at the assessment, as well as control for confounding factors. 

The strengths of the current study include a large sample size of validated non-smoking mothers (and their children followed up to two years of age) from the prospective cohort (REPRO_PL). In addition, repeated cotinine measurements throughout the whole pregnancy period and the questionnaire data obtained at the time of sample collection allows a more accurate exposure assessment. The series of detailed questionnaires also allow a reliable assessment of the covariates. Additionally, by restricting our population to healthy women, we were able to eliminate additional confounding variables. Finally, the study considered multiple aspects of child development.

However, the study has also some limitations. Firstly, even though the analysis included a variety of factors associated with passive smoking and child psychomotor development, it did not include the quality of the home environment, mother-child relationship, performing parental roles, or maternal IQ. SES can be considered a reliable proxy for most of these variables (IQ, job, and familial income). Marital status was associated to SES, with unmarried mothers having a SES significantly lower than married mothers. In addition, children of married and unmarried mothers did not differ as for cognitive, language, and motor Bayley scores at one-year and two-year assessments. As a matter of fact, the significant impact of numerous confounding factors in the analysis focusing on ETS and child neurodevelopment has been illuminated by Roza et al. [18]. Nevertheless, we did control (or considered for confounding effect) for maternal education, SES, number of siblings, and day care attendance as surrogate markers. Another limitation, which was also illuminated by Hsieh et al., is the possibility of a considerable overlap between prenatal and postnatal ETS exposure [12]. The Spearman correlation analysis was used to test the correlation between maternal (during the pregnancy period) and children’s (after birth) ETS exposure. Since the correlations between different indicators of maternal ETS exposure during pregnancy and maternal smoking after child birth were low (r < 0.2), the postnatal child ETS exposure was included as a confounding variable in the model. The negative impact of prenatal ETS exposure persisted even after adjusting for postnatal ETS. We also need to consider the limitation related with the use of standardized development assessment methods in early childhood. On one hand, they give the unique possibility for making comparisons between children based on “objective” developmental indices, which is crucial for epidemiological studies. On the other hand they are affected by measurement errors related to child’s general status at the time of examination and examiners’ approach [38]. Especially in the case of infants up to one year, reliable examination of neurodevelopment is challenging. Moreover, we also need to consider that the correlations obtained at the age of one year are not always confirmed at the age of two years. This might be the result of dynamic and, usually, at least to some extent, unbalanced development at this period of life which depends on environmental stimulation and attachment style, both depending on the characteristics of the child’s parents [39,40,41,42]. These factors that potentially affect child development were not controlled in our study. 

## 5. Conclusions

The current study demonstrates a negative impact of maternal ETS exposure during pregnancy on child psychomotor development, including cognitive, language, and motor abilities within the first two years of life. Our findings indicate that increased public awareness, health education, and intervention programs, simultaneously with adequate legislation, still require considerable improvement. Future studies are needed to determine whether the observed decrements in child psychomotor abilities have a long-term effect.

## Figures and Tables

**Table 1:** Spearman correlation between the Bayley test results assessments at one and two years of age.

Psychomotor Abilities		Assessment at the Age of One Year			Assessment at the Age of Two Years		
		Cognitive	Language	Motor	Cognitive	Language	Motor
**Assessments at the age of one year**	Cognitive	1					
	Language	0.28 **	1				
	Motor	0.30 **	0.43 **	1			
**Assessments at the age of two years**	Cognitive	0.09	0.04	0.15 *	1		
	Language	0.07	0.218 **	0.14 *	0.49 **	1	
	Motor	−0.03	0.14 *	0.15 *	0.06	0.37 **	1

For correlations within the assessment at one year of age *N* = 427; for correlations between assessments at the age of one and two years *N* = 258; for correlations within the assessment at the age of two *N* = 292. * *p* < 0.05; ** *p* < 0.01.

**Table 2:** Spearman correlation between indicators of exposure to environmental tobacco smoke (ETS).

ETS Exposure Variables	E1	E2	E3	E4	E5	E6	E7	E8	E9	E10	E11	E12	E13
**E1** Cotinine level ng/mL (1st trimester of pregnancy)	1	0.81 **	0.52 **	0.19 **	0.18 **	0.19 **	0.23 **	0.20 **	0.25 **	−0.01	0.02	0.16 **	0.19 **
**E2** Cotinine level ng/mL (2nd trimester of pregnancy)		1	0.73 **	0.25 **	0.24 **	0.20 **	0.24 **	0.20 **	0.25 **	0.09	0.10	0.24 **	0.22 **
**E3** Cotinine level ng/mL (3rd trimester of pregnancy)			1	0.14 **	0.11 *	0.10	0.18 **	0.14 **	0.17 **	0.09	0.05	0.14 **	0.09
**E4** Husband/partner smoking (1st trimester of pregnancy)				1	0.87 **	0.84 **	0.38 **	0.36 **	0.40 **	0.17 **	0.15 *	0.73 **	0.73 **
**E5** Husband/partner smoking (2nd trimester of pregnancy)					1	0.93 **	0.37 **	0.41 **	0.41 **	0.17 **	0.13 *	0.76 **	0.74 **
**E6**_Husband/partner smoking (3rd trimester of pregnancy						1	0.34 **	0.38 **	0.40 **	0.16 **	0.17 **	0.75 **	0.73 **
**E7** Smoking allowed at home (1st trimester of pregnancy)							1	0.75 **	0.73 **	0.17 **	0.18 **	0.39 **	0.36 **
**E8** Smoking allowed at home (2nd trimester of pregnancy)								1	0.81 **	0.19 **	0.19 **	0.40 **	0.41 **
**E9** Smoking allowed at home (3rd trimester of pregnancy)									1	0.17 **	0.25 **	0.39 **	0.43 **
**E10** Maternal smoking within one year after delivery										1	0.63 **	0.21 **	0.24 **
**E11** Maternal smoking within two year after delivery											1	0.06	0.17 **
**E12** Household smoking within one year after delivery												1	0.78 **
**E13** Household smoking within two year after delivery													1

* *p* < 0.05; ** *p* < 0.01.

**Table 3:** ETS exposure during pregnancy based on the cotinine level in saliva and child psychomotor development at one and two years of age. The multivariate linear regression model.

Cotinine	One-Year-Old Children β (*p*) *N* = 427			Two-Year-Old Children β (*p*) *N* = 292		
	Cognitive	Language	Motor	Cognitive	Language	Motor
1st trimester	−0.65 (0.60)	**−3.01 (0.02)**	−0.56 (0.69)	−3.05 (0.09)	**−3.81 (0.03)**	**−4.08 (0.02)**
2nd trimester	−1.06 (0.47)	**−3.55 (0.02)**	−0.43 (0.78)	**−5.33 (0.01)**	**−5.19 (0.009)**	**−5.19 (0.01)**
3rd trimester	1.04 (0.42)	−0.85 (0.52)	0.85 (0.54)	−1.26 (0.51)	0.11 (0.95)	**−4.38 (0.02)**

Cotinine value: 0 = less than 1.5 ng/mL, 1 = equal or greater than 1.5 ng/mL; Adjusted for: socio-economic status (0 = low, 1 = medium, 2 = high), child sex (0 = girl, 1 = boys), mother’s level of education (0 = below high school level, 1 = high school, 2 = university/college degree), mother’s age at delivery (continuous variable, years), examiner; For the assessment at the age of one year: 1st trimester *N* = 355, 2nd trimester = 365, 3rd trimester = 322; For the assessment at the age of two years: 1st trimester *N* = 251, 2nd trimester = 199, 3rd trimester = 232; Data are reported as β-beta coefficients (*p*-values). Regression coefficients significantly different from 0 are reported in bold.

**Table 4:** ETS exposure during pregnancy based on the selected indicators and child psychomotor development at the age of one and two years. The multivariate linear regression model.

Variables	One-Year Old Children β (*p*) *N* = 427			Two-Year Old Children β (*p*) *N* = 292		
	Cognitive	Language	Motor	Cognitive	Language	Motor
**1st trimester**						
Cotinine	−0.38 (0.77)	**−3.01 (0.03)**	0.41 (0.78)	−2.11 (0.29)	**−3.84 (0.05)**	**−3.85 (0.06)**
Husband smoking	**−3.28 (0.02)**	0.92 (0.54)	0.67 (0.68)	1.68 (0.43)	1.41 (0.50)	1.77 (0.42)
Smoking allowed at home	2.43 (0.15)	−0.86 (0.62)	0.09 (0.96)	−3.96 (0.11)	−1.48 (0.54)	−1.07 (0.67)
**2nd trimester**						
Cotinine	−0.53 (0.72)	**−4.14 (0.008)**	0.06 (0.97)	**−4.59 (0.05)**	**−6.27 (0.005)**	**−5.27 (0.02)**
Husband smoking	**−3.49 (0.05*)***	−0.20 (0.91)	−0.88 (0.64)	1.66 (0.53)	1.83 (0.46)	0.88 (0.74)
Smoking allowed at home	2.64 (0.20)	−0.22 (0.92)	0.70 (0.97)	−5.07 (0.09)	−3.16 (0.26)	−0.26 (0.93)
**3rd trimester**						
Cotinine	0.98 (0.46)	−1.16 (0.39)	0.69 (0.63)	0.37 (0.85)	0.23 (0.90)	**−4.16 (0.05)**
Husband smoking	**−3.65 (0.02)**	−1.34 (0.40)	−1.60 (0.35)	0.93 (0.68)	1.71 (0.44)	1.69 (0.49)
Smoking allowed at home	2.02 (0.29)	0.16 (0.93)	0.65 (0.75)	−4.96 (0.07)	−3.01 (0.26)	−1.72 (0.55)

Prenatal exposure indicators: cotinine value (0 = less than 1.5 ng/mL, 1 = equal or greater 1.5 ng/mL); husband smoking in the specified trimester of pregnancy (0 = No, 1 = Yes); smoking allowed at home in the specified trimester of pregnancy (0 = No, 1 = Yes). Adjusted for: socio-economic status (0 = low, 1 = medium, 2 = high), child sex (0 = girl, 1 = boys), mother’s level of education (0 = below high school level, 1 = high school, 2 = university/college degree), mother’s age at delivery (continuous variable, years), maternal smoking within one year after delivery (0 = No, 1 = Yes), examiner; For the assessment at the age of 1-year: 1st trimester *N* = 342, 2nd trimester = 253, 3rd trimester = 310; For the assessment at the age of 2-year: 1st trimester *N* = 221, 2nd trimester = 179, 3rd trimester = 211; Data are reported as β-beta coefficients (*p*-values). Regression coefficients significantly different from 0 are reported in bold.

## Supplementary Materials

The following are available online at www.mdpi.com/1660-4601/14/7/796/S1, Table S1: Child and parental characteristics; Table S2: Characteristics of exposure and outcome variables; Table S3: ETS exposure during pregnancy based on selected indicators and psychomotor development at one and two years. Multiple linear regression model; Table S4: Socio-economic status: Low/Medium. ETS exposure during pregnancy based on the cotinine level in saliva and child psychomotor development at one and two years of age. Multiple linear regression model; Table S5: Socio-economic status: High. ETS exposure during pregnancy based on the cotinine level in saliva and child psychomotor development at one and two years of age. Multiple linear regression model.
