# Supplementary material for: Environmental Tobacco Smoke Exposure during Pregnancy and Child Neurodevelopment
Source: Int J Environ Res Public Health. 2017 Jul 17;14(7):796. doi: 10.3390/ijerph14070796 (PMC5551234; doi:10.3390/ijerph14070796)
Supplement: Supplementary file 1 [file ijerph-14-00796-s001.pdf]

Supplementary Materials: Environmental Tobacco Smoke Exposure during Pregnancy and Child Neurodevelopment

Kinga Polanska, Anna Krol, Dorota Merecz-Kot, Danuta Ligocka, Karolina Mikolajewska, Fiorino Mirabella, Flavia Chiarotti, Gemma Calamandrei and Wojciech Hanke

Table S1. Child and parental characteristics (N = 461).

**Characteristic**	**Respondents**	
Qualitative Variables	*N*	%
Sex		
Boys	214	46.4
Girls	247	53.6
Breastfeeding		
No	45	9.8
Yes	416	90.2
Day care attendance at one year of age		
Yes	31	7.7
No	371	92.3
Day care attendance at two years of age		
Yes	64	23.7
No	206	76.3
Mode of delivery		
Cesarean	153	37.1
Vaginal	259	62.9
Parity		
0	248	53.8
≥1	213	46.2
Marital status		
Married	356	77.9
Unmarried	101	22.1
Maternal education		
Below high school level	10	2.2
High school	136	29.6
University/college degree	314	68.3
Paternal education		
Below high school level	16	3.5
High school	235	51.8
University/college degree	203	44.7
Socioeconomic status		
Low	39	8.6
Medium	323	70.1
High	92	20.3
Alcohol		
Yes	31	7.7
No	373	92.3
Quantitative variables	Mean	SD
Gestational age (weeks)	39.2	1.4
Birth weight (Kg)	3.4	0.5
Maternal age at delivery	29.2	4.3
Paternal age at delivery	31.0	5.4
Maternal pre-pregnancy BMI (kg/m^2)	22.4	3.7

Missing data 0–10.6%.

Table S2. Characteristics of exposure and outcome variables (*N* = 461).

Characteristic	Respondents		
Exposure Variables	*N*	%	% > LOQ
ETS exposure during pregnancy based on cotinine cut-off (1.5 ng/mL)			
1st trimester of pregnancy	128/379	33.8	93.6
2nd trimester of pregnancy	100/286	35.0	96.9
3rd trimester of pregnancy	114/336	33.9	95.9
Husband/partner smoking			
1st trimester of pregnancy	136/449	30.3	
2nd trimester of pregnancy	124/438	28.3	
3rd trimester of pregnancy	113/419	27.0	
Smoking allowed at your home			
1st trimester of pregnancy	95/450	21.1	
2nd trimester of pregnancy	81/439	18.5	
3rd trimester of pregnancy	73/419	17.4	
Maternal smoking within one year after delivery	38/388	9.8	
Maternal smoking within two years after delivery	28/262	10.7	
Cotinine level in child urine collected at one year of age (ng/mL) GM ± SD; *N* = 183	2.4	5.2	79.2
Cotinine level in child urine collected at two years of age (ng/mL) GM ± SD; *N* = 140	5.8	6.7	82.9
Outcome Variables	Mean	SD	
Neurodevelopment score (Bayley 3rd edition)			
Cognitive (one year)	106.6	10.8	
Language (one year)	108.3	12.7	
Motor (one year)	105.0	13.3	
Cognitive (two years)	110.4	16.1	
Language (two years)	101.9	12.8	
Motor (two years)	112.0	14.6	

LOQ: limit of quantification.

Table S3. ETS exposure during pregnancy based on selected indicators and psychomotor development at one and two years. Multiple linear regression model.

Variables	One-Year Old Children β (*p*)			Two-Year Old Children β (*p*)		
	Cognitive	Language	Motor	Cognitive	Language	Motor
First trimester						
Cotinine	–2.59 (0.16)	**–5.05 (0.01)**	–0.64 (0.75)	**–4.55 (0.04)**	–3.90 (0.12)	**–5.44 (0.05)**
Husband smoking	**–4.89 (0.02)**	–0.85 (0.69)	0.72 (0.76)	3.71 (0.13)	–1.10 (0.69)	1.73 (0.56)
Smoking allowed at home	3.04 (0.24)	–0.45 (0.86)	–1.76 (0.53)	–0.83 (0.78)	3.70 (0.27)	–0.77 (0.83)
Second trimester						
Cotinine	**–4.81 (0.02)**	**–5.11 (0.01)**	–0.06 (0.98)	**–7.06 (0.01)**	**–7.15 (0.01)**	–5.69 (0.08)
Husband smoking	**–6.59 (0.01)**	–3.55 (0.15)	–3.03 (0.26)	1.34 (0.66)	0.42 (0.90)	–0.84 (0.81)
Smoking allowed at home	**6.53 (0.03)**	3.85 (0.17)	1.53 (0.62)	0.36 (0.09)	2.18 (0.52)	2.35 (0.54)
Third trimester						
Cotinine	–3.19 (0.07)	**–3.71 (0.04)**	–1.32 (0.48)	0.48 (0.84)	2.45 (0.32)	–1.12 (0.68)
Husband smoking	**–6.55 (0.004)**	–3.21 (0.16)	–3.63 (0.12)	–0.69 (0.79)	–1.64 (0.56)	1.58 (0.62)
Smoking allowed at home	4.79 (0.07)	2.79 (0.30)	2.35 (0.41)	2.66 (0.46)	6.37 (0.09)	1.12 (0.79)

Prenatal exposure indicators: cotinine (0 = less than 1.5 ng/mL, 1 = equal or greater 1.5 ng/mL); husband smoking in the trimester of pregnancy (0 = No, 1 = Yes); smoking allowed at home in the trimester of pregnancy (0 = No, 1 = Yes). **Adjusted for**: socio-economic status (0 = low, 1 = medium, 2 = high), child sex (0 = girl, 1 = boys), mother's level of education (0 = below high school level, 1 = high school, 2 = university/college degree), mother's age at delivery (continuous variable, years), cotinine in child urine (ng/mL), Examiner. For the assessment at the age of one year: 1st trimester *N* = 149; 2nd trimester = 120; 3rd trimester = 143. For the assessment at the age of two years: 1st trimester *N* = 116; 2nd trimester = 94; 3rd trimester = 112. Data is reported as β—beta coefficients (*p*-values). Regression coefficients significantly different from 0 are reported in bold.

Table S4. Socio-economic status: Low/Medium. ETS exposure during pregnancy based on the cotinine level in saliva and child psychomotor development at one- and two-years of age. Multiple linear regression model.

**Cotinine**	**One-Year Old Children β (*p*)**			**Two-Year Old Children β (*p*)**		
	**Cognitive**	**Language**	**Motor**	**Cognitive**	**Language**	**Motor**
**1st trimester**	–0.32 (0.82)	**–3.76 (0.01)**	–0.37 (0.80)	–2.76 (0.18)	**–5.34 (0.01)**	**–4.40 (0.03)**
**2nd trimester**	–0.928 (0.57)	**–3.59 (0.03)**	–0.22 (0.89)	**–4.78 (0.04)**	**–5.66 (0.01)**	–3.90 (0.09)
**3rd trimester**	1.54 (0.29)	–0.65 (0.66)	0.99 (0.51)	–0.35 (0.87)	0.65 (0.76)	–3.63 (0.09)

Cotinine value: 0 = less than 1.5 ng/mL, 1 = equal to or greater than 1.5 ng/mL. **Adjusted for**: Child sex (0 = girl, 1 = boys), mother's level of education (0 = below high school level, 1 = high school, 2 = university/college degree), mother's age at delivery (continuous variable, years), Examiner. For the assessment at the age of one-year: 1st trimester = 284; 2nd trimester = 221; 3rd trimester = 256. For the assessment at the age of two-years: 1st trimester = 209; 2nd trimester = 170; 3rd trimester = 193. Data is reported as β (beta coefficients) (*p*-values). Regression coefficients significantly different from 0 are reported in bold.

Table S5. Socio-economic status: High. ETS exposure during pregnancy based on cotinine level in saliva and child psychomotor development at one- and two-years of age. Multiple linear regression model.

**Cotinine**	**One-Year Old Children β (*p*)**			**Two-Year Old Children β (*p*)**		
	**Cognitive**	**Language**	**Motor**	**Cognitive**	**Language**	**Motor**
1st trimester	–0.74 (0.79)	1.41 (0.66)	2.23 (0.58)	–2.53 (0.45)	2.50 (0.47)	–6.50 (0.16)
2nd trimester	–0.20 (0.95)	–3.85 (0.30)	0.69 (0.89)	–1.56 (0.72)	–1.50 (0.67)	–8.46 (0.10)
3rd trimester	–1.44 (0.70)	–1.35 (0.65)	0.27 (0.94)	–0.89 (0.82)	0.21 (0.95)	–5.75 (0.18)

Cotinine value: 0 = less than 1.5 ng/mL, 1 = equal to or greater than 1.5 ng/mL. **Adjusted for**: Child sex (0 = girl, 1 = boys), mother's level of education (0 = below high school level, 1 = high school, 2 = university/college degree), mother's age at delivery (continuous variable, years), Examiner. For the assessment at the age of one-year: 1st trimester = 71; 2nd trimester = 44; 3rd trimester = 66. For the assessment at the age of two-years: 1st trimester = 42; 2nd trimester = 29; 3rd trimester = 39. Data is reported as β (beta coefficients) (*p*-values).

© 2017 by the authors; licensee MDPI, Basel, Switzerland. This article is an open access article distributed under the terms and conditions of the Creative Commons by Attribution (CC-BY) license (http://creativecommons.org/licenses/by/4.0/).
